# “PrEP should be available all the time and everywhere”: A qualitative assessment of family planning and PrEP integration in Lesotho

**DOI:** 10.3389/frph.2022.981845

**Published:** 2022-09-06

**Authors:** Nyane-Matebello Nonyana, Manthomeng Matete, Elena Lebetkin, Irina Yacobson, Molly Strachan, Makeneiloe Anastasia Ramapepe, Morrisa Malkin, Tafadzwa Chakare

**Affiliations:** ^1^Jhpiego, Maseru, Lesotho; ^2^FHI 360, Durham, NC, United States; ^3^Jhpiego, Baltimore, MD, United States

**Keywords:** family planning, pre-exposure prophylaxis (PrEP), integration, HIV prevention, integrated services, Sub-Saharan Africa

## Abstract

**Background:**

Lesotho has a high HIV burden, with women disproportionately affected. Increased access points for HIV prevention services, including oral pre-exposure prophylaxis (PrEP), should be considered. Using family planning (FP) settings for PrEP services may contribute to greater uptake of HIV prevention methods.

**Methodology:**

This formative qualitative assessment was conducted in Maseru District, Lesotho and included in-depth interviews with 15 key informants, 10 FP providers in public facilities and community sites, and 15 FP and PrEP clients from facility and community sites. Interviews were audio recorded and in lieu of producing transcripts, teams completed semi-structured data extraction tables after each interview. Findings were compiled and synthesized by participant group into matrices and themes identified through deductive and inductive analysis.

**Results:**

Policy makers were generally supportive of integration but felt hampered by lack of integration policies and separation of HIV and FP departments at Ministry of Health. Funders stressed the need for coordination among partners to avoid duplication of efforts. Partners felt clients would be interested in PrEP/FP integration and that PrEP demand creation and education were crucial needs. Most providers supported integration, stressing the potential benefit to clients. Barriers discussed included heavy workloads, staff shortages, training needs, separate registers for FP and PrEP, and commodity stock-outs. Providers discussed strengthening integrated services through training, increasing staffing, having job aids and guidelines, merging the FP and PrEP registers, and marketing services together to create demand for both. Clients were overwhelmingly willing to have longer visits to receive comprehensive services and were supportive of receiving PrEP services from FP providers. Clients not using PrEP expressed willingness and interest to use. Clients' suggestions for successful integration included consulting with youth, conducting community outreaches, and improving provider availability.

**Conclusions:**

Existing FP platforms are established and well-utilized; thus providing opportunities for integrating PrEP. This assessment found support across all groups of respondents for providing PrEP within FP settings and identified a number of facilitators and barriers to integration. As PrEP rollout is relatively nascent in many countries, deepening the evidence base early will enable the utilization of findings to build stronger integrated programs with wider coverage.

## Introduction

The human immunodeficiency virus (HIV) prevalence is high in Lesotho at 22% of the adult population ages 15–49 (1, 2). Despite the high HIV burden, Lesotho has demonstrated significant progress in achieving epidemic control by recently meeting or exceeding the 90–90–90 targets set by the Joint United Nations Programme on HIV/AIDS (UNAIDS), with 90% of all people living with HIV knowing their status, 97% of people diagnosed being on antiretroviral therapy (ART), and 92% of people on ART achieving viral suppression (1). Despite notable achievements, gaps such as the marked disparities in HIV prevalence when data are stratified by age and sex remain (1). The most pronounced differences are in the 25–29 year-old age group where the HIV prevalence is 7% in males and 28% in females and the 30–34 year-old age group with HIV prevalence being 19% among males and 41% among females (1). Increasing access to HIV prevention services such as oral pre-exposure prophylaxis (PrEP), especially among populations with high HIV prevalence, including young women, is a programmatic intervention that should be considered in order to achieve epidemic control. PrEP has been shown to be a highly effective and safe HIV prevention method for multiple populations (3). Evidence from a number of countries has shown that integrating HIV services, such as counseling and testing services, into family planning (FP) settings is feasible and acceptable (4–7). However, the evidence on integrating PrEP and FP services specifically is limited and shows that integration is feasible under the context of studies and programs with mixed evidence on PrEP uptake and adherence (8–12). The use of contraceptive methods is high in Lesotho with 60% of married women using a modern method, though the method mix is heavily skewed toward condoms, progestin-only injectables, and oral contraceptive pills (OCPs) (13). With the high use of contraception, most of which is obtained at public and private sector facilities (13), providing PrEP in FP settings may be a feasible strategy for making effective HIV prevention services more accessible to an at-risk population.

To some extent, national policies and guidelines in Lesotho address PrEP provision and integration into FP. The *Addendum to the National Guidelines on the Use of Antiretroviral Therapy for HIV Prevention and Treatment*, issued in July 2019, provides the clearest recommendation that PrEP should be integrated into various services at multiple service delivery points, including within FP clinics (14). Notably, the 2017 *Lesotho National Family Planning Guidelines for Health Service Providers* do not specifically address integrating PrEP into FP services, while recommending that FP be integrated into a variety of other services, including HIV services (15).

A previous landscape analysis to assess the degree of PrEP roll-out and integration into FP services was conducted in 2020 in seven Sub-Saharan African countries, including Lesotho. The analysis found fairly limited PrEP roll-out in Lesotho with PrEP services available mostly through HIV services at the facility and community levels (16). Building on the landscape analysis, we conducted this qualitative assessment in Maseru District where PrEP provision is ongoing at both the U.S. Agency for International Development (USAID)-supported facilities and at community-level sites for the U.S. President's Emergency Plan for AIDS Relief (PEPFAR) Determined, Resilient, Empowered, AIDS-free, Mentored and Safe (DREAMS) initiative. DREAMS is an initiative aimed at prioritizing the health and wellbeing of adolescent girls and young women (AGYW) aged 10–24 years through multiple layers of interventions including PrEP and FP provision. In Lesotho, DREAMS is implemented in collaboration with five PEPFAR-supported Implementing Partners—Jhpiego, PSI, Elizabeth Glaser Pediatric AIDS Foundation (EGPAF), Karabo-Ea-Bophelo (KB), and Mothers-to-Mothers (M2M). Jhpiego, PSI, and KB provide services at the community level and EGPAF and M2M provide services at the facility level. Our assessment was conducted in EGPAF-supported facility sites and Jhpiego community-level sites in Maseru District to understand the feasibility and acceptability of integrating PrEP into FP services where the same FP provider is able to offer both services, and to inform planning and advocacy for integrated services. The assessment was funded by PEPFAR and USAID.

## Methods

This formative qualitative assessment consisted of in-depth interviews (IDIs) conducted in English or Sesotho, depending upon the preference of the respondent. Data were collected from January through March 2021 in the urban Maseru District. Respondents were purposively selected and the sample size for each group was based on time, resource availability, and previous literature on qualitative sample size (17, 18). Key informants were identified based on expert knowledge and were eligible for inclusion if they had knowledge of FP and PrEP programming, funding, and/or policy due to their employment. We conducted IDIs with a total of 15 key informants, representing FP and/or HIV policymakers (n = 5), FP and/or HIV implementing partners (n = 5), and funders (n = 5). Providers were eligible for inclusion if they provided FP or FP/HIV services in a facility or community setting. We liaised with the implementing partners to identify eligible providers—EGPAF for facility sites and Jhpiego for community sites. IDIs were conducted with 10 FP providers from the facility level (n = 5) and community level at DREAMS sites (n = 5). Finally, providers at facilities and community sites were engaged to read a script to clients seeking FP and PrEP and identify clients who were eligible and interested in participating in the assessment. Clients were then contacted by trained research assistants who confirmed eligibility, and consenting clients were interviewed. IDIs were conducted with 7 FP clients who indicated they would consider using PrEP (n = 4 from the facility and n = 3 from the community) and 8 PrEP clients who are either using FP now or who indicated they would consider using FP in the future (n = 4 from facility and n = 4 from community).

Interview guides were informed by previous work by Bhavaraju et al. (16) which identified essential elements required to support effective integration of PrEP into FP services. Prior to study implementation, the interview guides underwent technical reviews from multiple technical experts in Lesotho and the United States (US) to ensure the guides were technically sound and appropriate for the local context. Key informant IDI topics included barriers, facilitators, and gaps in national policies and guidelines related to FP and HIV/PrEP integration, feasibility and acceptability of integrating PrEP into FP services, procurement and supply chain, demand creation, provider training, and recommendations to improve programs. Provider IDI guides explored the level of integration occurring in facilities, feasibility and acceptability of integration, barriers and facilitators to integrated services, referral practices, record-keeping and reporting requirements, training received and needed to provide integrated services, demand creation, procurement and supply chain, and recommendation to improve programs. IDIs with clients explored experiences with FP and PrEP use and how these experiences can be improved, familiarity with PrEP, acceptability of service integration, and barriers and facilitators to integrated services.

Interviews were conducted either in-person or *via* phone depending on COVID-19 safety regulations at the time of the interview. Two trained research assistants participated in all interviews—one research assistant conducted the interview, and the other audio recorded the interviews and took detailed notes. Research assistants were trained on the importance of developing rapport with participants to ensure the interviews were rich with detail and allowed for participants to feel comfortable bringing up any additional areas of interest or themes not covered in the interview guide during the interview. In lieu of producing transcripts, research assistants collectively completed a semi-structured data extraction sheet after each interview. The data extraction sheets included sections to record verbatim comments from participants which were obtained from the audio recordings. For each participant group, we used an analysis matrix comprised of codes (deductive themes in matrix columns) based on the interview guides as well as inductive themes that emerged during analysis. Themes were synthesized by participant group and then compared across all participant groups to identify similarities as well as variations in perceptions on the feasibility and acceptability of integration.

From our data we observed four main themes that influence the acceptability and feasibility of integrating PrEP into FP services: enabling environment, perceptions of acceptability, service readiness, and service utilization and willingness to use PrEP. Enabling environment factors included supply chain, infrastructure, and governmental commitment and support such as training resources, policies and guidelines, and intragovernmental cooperation. Perceptions of acceptability encompassed attitudes, preferences, and beliefs about the acceptability of integrated services. Service readiness comprised provider and key informant perceptions of factors that support or hinder the ability of providers and facilities to deliver integrated services. Finally, service utilization and willingness to use PrEP looked at client-level factors such as social support, current service use and experiences, and community norms (Table 1).

**Table 1:** Themes explored (by participant group).

**Respondents**	**Key informants**	**FP providers**	**FP clients**	**PrEP clients**
**Themes**
Enabling environment	X	X		
Perceptions of acceptability	X	X	X	X
Service provision and readiness	X	X		
Service utilization and willingness to use PrEP			X	X

This assessment was reviewed and approved by FHI 360's Protection of Human Subjects Committee, the Johns Hopkins School of Public Health Institutional Review Board, and the Lesotho Ministry of Health Research and Ethics Committee. All participants were adults 18-years-old or older and were administered informed consent and verbally provided their consent to participate in the assessment.

### Positionality statement

Prior to presenting results, the team acknowledges the bias brought to the assessment as a group of highly educated individuals with varied nationalities, experiences, and worldviews which affect our ability to remain free of bias. The team was led by highly experienced and trained researchers, program implementers, and clinical staff who are Basotho and live and work in Lesotho along with an equally experienced US-based study team. We also acknowledge the additional privilege of the US-based study team who has benefited from living and working in a high-income country environment. Finally, we would like to acknowledge that like all for all qualitative research, researchers are not unbiased and this affects the interpretation of the data.

## Results

### Characteristics of participants

#### Key informants

Key informants represented three stakeholder groups—policymakers, implementing partners, and funders. We interviewed five policymakers from the Ministry of Health who served in various roles in both FP and HIV fields, with experience ranging from four to more than 30 years. Five representatives from implementing partner organizations that we interviewed included those from EGPAF, PSI, Baylor College of Medicine Children's Foundation Lesotho, the Christian Health Association of Lesotho (CHAL), and Lesotho Planned Parenthood Association (LPPA). All were either nurses or doctors with five to 20 years of experience who managed/coordinated FP and/or HIV services for their organizations. Finally, we interviewed five representatives from HIV and FP donor agencies, including USAID, United Nations Population Fund (UNFPA), UNICEF, Global Fund/Project Management Unit (PMU), and the Centers for Disease Control and Prevention (CDC). The donor interviewees' experiences were varied and included coordination of sexual and reproductive health (SRH) programs, coordination and implementation of laboratory strategies, supporting supply chain activities, monitoring performance of grantees and ensuring effective implementation of monitoring and evaluation (M&E) activities. All were highly experienced in the FP field (with an average of 9 years) and/or the HIV field (with average of 12 years).

#### FP providers

We interviewed five community-level FP providers and five facility-level FP providers. Their experience with offering FP and other SRH services varied greatly from 10 months to 12 years. The community-level providers were part of the DREAMS program, serving AGYW. In addition to FP services, four also offered HIV counseling and testing services, three provided ART, and one provided PrEP. All of the facility-level providers were MOH employees and all five offered at least one other service in addition to FP, including cancer screening, antenatal care, ART, PrEP, prevention of mother-to-child transmission (PMTCT), and pediatric care.

#### FP clients who would consider using PrEP

We interviewed a total of seven female FP clients: four from the facility level (from two different facilities) and three from the community level (from two different Jhpiego community resource centers implementing the DREAMS program). Clients ranged from 21- to 31-years-old (average 25 years), two had no children and five had one child; four clients were married. The women were all in school ranging from secondary school to tertiary school, including university.

#### PrEP clients who are using FP or would consider future FP use

We interviewed a total of eight current PrEP clients: four from the facility level (from two different facilities), and four from the community level (from two different DREAMS sites). Clients ranged from 21 to 39 years-old (average 27 years), six had at least one child (maximum 4 children) and two had no children; the majority of respondents were married (n = 6). Education level attained ranged from high school to tertiary education including college and university.

### Enabling environment

All policymakers interviewed felt that the government of Lesotho has shown some political commitment and leadership toward integration of services by allocating funds toward in-country capacity building for health professionals and procurement of antiretroviral (ARV) medications and FP commodities. They indicated that the government contributes 70% toward procurement of ARVs, including PrEP, and 50% toward FP commodities while Global Fund contributes 30% toward ARVs and UNFPA contributes 50% toward FP commodities. Some pointed out that PEPFAR funds are dedicated strictly for HIV interventions, with no commodities procured.

Although most key informants acknowledged that no policies guiding integration of services exist, some implementing partners reflected on two guidelines that promote integration: *The Addendum to the National Guidelines on the Use of Antiretroviral Therapy for HIV Prevention and Treatment* (14) stating that PrEP should be integrated into various service points including FP, and *The Lesotho National Family Planning Guidelines for Health Service Providers* (15) which does not specifically address PrEP or PrEP/FP integration but does recommend integrating FP into a range of other services, including HIV services. Key informants explained that the guidelines are reviewed every 2 years through consultation meetings with relevant stakeholders at district level including public health nurses, service providers, and community-based organizations (CBOs); they felt that these meetings could also be used to develop/strengthen policies related to integration. Policy dissemination occurs through validation meetings and subsequent distribution of hard copies to facilities, sometimes with the help of implementing partners.

Nevertheless, one key informant respondent believed that dissemination to districts and front-line providers is not well implemented, and monitoring of policy implementation needs extra attention. This finding was echoed by providers who reported limited availability of national FP guidelines on site. Two community-level providers reported that the guidelines are available, one was not sure, one said they were unavailable, and one did not respond. Only one community-based provider had received training on the latest FP guidelines. At the facility level, two providers reported that FP guidelines are not available, and that they had no training on the most recent guidelines; another said that while guidelines are not available, she/he attended a training workshop on guideline implementation, and two responded that they do have FP guidelines at their facility and received training on the content. All providers reported either not having PrEP guidelines on site or not knowing if the guidelines were on site and only one community-level provider reported receiving PrEP training multiple years ago (2018).

Key informants highlighted programmatic constraints that could hinder integration of PrEP into FP services and are related to the way services are managed and funded. Because the two services are managed under different departments within the Ministry of Health (MOH)—PrEP services are under the Department of Disease Control while FP services are under the Department of Family Division—one respondent felt that getting the two departments to work together will require effort, saying:

“*So, getting those two to the table is so hard…we still have a bit of advocacy to do around that*.”

-Funder

In addition, implementing and maintaining integration of services will be challenging as most HIV interventions have limited are seen as a mandate for implementing partners with limited MOH ownership. As implementing partners are donor funded, once the funding ends, the program usually ends.

“*When donors pull down their support, programs fall to the ground*.”

-Implementing Partner

This sentiment was echoed:

“*... because most of the time we are supported by partners, when the project ends, we struggle to continue.”*

-Policymaker

Additional challenges with donor funding discussed included vertical funding silos in which funds are provided for specific service, such as HIV, which does not allow for integrated programming. Most key informant respondents felt coordination of PrEP/FP integration should be led by the MOH through the Health Planning and Statistics Department (HPSD) with support of funders like PEPFAR and UN agencies. They also acknowledged that there are no specific technical working groups (TWGs) or communities of practice for integration of PrEP into FP services within the MOH. Hence, several respondents recommended incorporating PrEP/FP integration into existing TWGs (e.g., HIV/tuberculous TWG and Sexual and Reproductive Health TWG). One respondent stated:

“*We have many technical working groups, so the existing technical groups can incorporate these two and integrate them. The technical working groups are made of the same people discussing different areas and topics, so they need to integrate, not form a new one.”*

-Funder

Another respondent indicated that PrEP/FP integration could also be discussed in and Village Health Worker forums and Health Centre Committees (HCCs). HCCs are responsible for overseeing the activities of health centers and are made up of representatives from different community groups within the catchment area of a facility such as AGYWs, chiefs, councilors, traditional doctors, Public Health Nurses, and Village Health Workers. A subset of HCCs, Village Health Worker forum are made up of women who have been trained on how to support and provide certain health services at the community level.

Some key informants discussed that facilities are already providing what could be considered integrated services through the “supermarket approach” whereby services are offered under one roof and on the same day, but by different providers. However, this approach differs from the integration approach explored in our assessment in which one provider is able to offer multiple services at a single visit. Currently, collaboration between FP and HIV providers to offer more holistic services is somewhat inconsistent. Three providers at the facility level reported some collaboration, including information sharing and joint planning, with the other two saying they are not aware of collaboration. At the community level, two FP providers said that they often plan outreach and mobilization activities together with HIV providers, but three said that it is possible that such collaboration occurs at the managerial level, but it does not occur at the provider level, with one saying that “*collaboration ends at sharing the facility*.”

Challenges in service provision were discussed by key informants and providers including a shortage of staff, limiting the number of services that can be provided, as well as providers' ability to take on more tasks. Key informants felt that providers are burdened by multiple reporting forms required every time they see a client. Providers expressed similar concerns including increased paperwork noting that FP and PrEP client registers are separate and integrated services would require reporting in multiple registers.

“*As for providers it will be too much workload as there will be many registers to use*.”

-Facility-level provider

Having limited access to FP services in some areas was also mentioned as a barrier by key informants (e.g., areas served by Catholic facilities which do not offer FP services outside of referrals). Other constraints discussed by key informants and providers included insufficient infrastructure (e.g., unavailability of private space for adolescent corners), limited provider training (both pre-service and in-service) including lack of proper PrEP training, lack of guidelines and job aids, frequent commodity stock-outs, and weak referral systems. However, some key informants disagreed that the infrastructure was inadequate, indicating that the country's infrastructure is sufficient to provide integrated services including an allocated space for FP services in all health facilities.

Additionally, community-level providers acknowledged possible client-side barriers, such as longer queues if providers are spending more time with each client receiving integrated services. Other client-side barriers mentioned by providers included clients experiencing “*information overload*” and a general concern about clients not utilizing integrated services unless adequate sensitization takes place due to misconceptions about PrEP.

Four FP providers (three at the community level and one at the facility level) expressed some specific concerns about increased workload due to PrEP/FP integration. One provider felt that this should be addressed by salary increases and another worried that having one provider offering two services may lead to job loss for others.

Providers discussed potential ways to overcome barriers to providing integrated services. They included building capacity of the existing staff through provider training on PrEP as well as having practical job aids and clear guidelines for integration, adding more staff to compensate for higher workload, designing new client forms that include both FP and PrEP, merging registers, addressing stock-outs to ensure adequate supply of both FP methods and PrEP commodities, marketing both services together, and including both in mobilization campaigns. Community-based providers also mentioned rebranding their tents to make it clear that they offer both FP and PrEP and using mobile clinics to reach clients in remote areas with both services.

### Perceptions of acceptability

Many key informant respondents, especially the implementing partners and funders, felt the integration of PrEP into FP services would be well received by AGYWs, married women, factory workers, and key populations (e.g., female sex workers) as it would reduce both their visits to health facilities and time spent queuing for different services. A respondent stated:

“*I don't have any concerns with integration of PrEP into FP services because the benefits are clear. We are averting new infections and unplanned pregnancies, so the integration reduces the burden for the beneficiaries to have to come to the clinic twice [which involves] issues of transport and time, and also reduces clinic congestion because people won't be coming on separate days for two services. They will have them in one day—give them two to three months' supplies and they go.”*

-Funder

Expressing similar views, all of the FP clients we interviewed said that if they were to use PrEP, they would prefer to get both PrEP and FP services at the same time and from a single provider. Women felt that this integrated approach would help encourage continuation of both FP and PrEP, reduce the number of queues they were required to wait in, and that the number of health care visits would be reduced thus saving time and money. Also, women felt that this approach would allow them to build a relationship with a single provider which may result in better trust and allow for more holistic client-centered care.

Providers also believed that integrating PrEP into FP services would benefit their clients. Eight out of 10 FP providers (three at community level and five at facility level) felt that integrating services would reduce the number of client visits and eliminate the need for clients to wait in two separate queues. This would save time as well as money clients would otherwise spend on transportation or lose by taking time off work. As one of the providers described:

“*Those who need both services would love to get them at one point with the main driver being the waiting time.”*

-Community-level provider

Some providers felt that integrated services may improve continuation and adherence by offering refills and counseling for both services at the same time, as well as reduce number of incomplete referrals.

However, some providers thought that offering PrEP as part of the FP visit is not necessary when both services can be obtained within the same facility or community site. They also felt that having the same provider offering both services may affect service quality, and that for integration to be effective, providers' workload should be considered as iterated by a provider:

“*Integration would add too much workload, paperwork, and reporting.”*

-Community-level provider

Some key informants felt similarly and were concerned that the waiting time may increase with service integration as the providers will spend more time with each client in order to provide integrated services. A respondent expressed concern, saying:

“ …* if there is inadequate staff, they will complain of the waiting time; they will have to wait for a longer time for one person in the consultation room to get services*.”

-Policymaker

However, most FP and PrEP clients interviewed felt differently as multiple respondents said that they would prefer receiving integrated PrEP/FP services regardless of how much time it could take, as expressed below:

”*[I] would love to get comprehensive services even if it means spending the whole day at facility.“*

-FP client at facility level, 26-years-old married with 1 child

Only two PrEP clients indicated that they would rather see separate providers for FP and for PrEP, with one of them pointing out that it would not be possible for her to consistently get both services in one visit as she receives norethisterone enanthate (NET-EN) reinjection every 2 months, but PrEP resupply is given only for 1 month at a time. One PrEP client expressed her support for the current “supermarket approach” in which she is able to receive both services during one visit to the health facility, though from different providers.

Key informants also expressed concern that providers may be unhappy with the additional workload resulting from having to offer integrated services, though respondents believed that providers would adapt as the practice is rolled out. Almost half of the key informant respondents indicated that socio-cultural norms could play a pivotal role in the acceptance of integrated services by clients as some rural women may require consent from their spouses to seek health services; hence, male engagement in the initial stages of PrEP/FP integration will be crucial to success. Opinions about acceptability of PrEP/FP integration by communities differed across respondents. Among policymakers and implementing partners, some felt that communities may perceive PrEP provision to AGYW as something that will promote promiscuous behavior; others disagreed and thought communities would welcome integration of PrEP into FP services. Among funders, only one shared the view that communities will accept integrated services and the remainder felt that misconceptions about PrEP would hamper integration.

Clients also noted the role of community norms in service provision and provided suggestions for how to successfully roll out PrEP/FP integration including community outreach with clear messaging about the services available and education about PrEP as it is not a well-known drug and is commonly confused with ART. One respondent provided a quote which summarized the suggestions:

”*My suggestion is that it should be available all the time and everywhere, and also teach people about PrEP because people believe it is ART, so the containers should be different.“*

-PrEP client at facility level, 32-years-old married with 1 child

Additionally, clients recommended consulting with underserved groups, such as AGYW, to better understand and respond to their needs around integrated services.

### Service readiness and provision

Almost all key informants felt the country was well positioned to leverage existing structures to provide PrEP/FP integrated services. They highlighted opportunities for integration in ongoing services such as by volunteer health workers (VHW) who already provide refills for OCPs in the community and could be trained to provide PrEP refills. They also noted that having some HIV services at facility level already integrated with SRH/FP services makes it easier to add PrEP. A respondent summarized the opportunity well:

“*One of the strengths is that there are already existing integration services from which they can benchmark; the only thing to do would be to identify how to expand it to cover more services that are being integrated. In the SRH program we already have HIV/SRH integration, so PrEP being the new thing that is coming, I think it is going to be easy to incorporate it.”*

*-Implementing Partner*

Moreover, some key informants mentioned that the country has a national pool of trainers who can be called upon to provide in-service training for FP providers on either PrEP or PrEP/FP integration of services. Training on PrEP had not occurred among facility-based providers as none of the five facility-based FP providers reported receiving PrEP training. However, PrEP training was much more common among community-level FP providers with four out of five having at least some basic training, lasting from 1 hour to 1 day. The remaining provider expressed interest in being trained on PrEP and felt that this would be important to better serve the needs of their client base. This provider said:

“*Because I work with AGYW clients and they are a population group that makes a thorough research before they come for services, it is imperative that as a provider, I should as well empower myself and acquire more knowledge”*.

-Community-level provider

Some key informants also mentioned that FP commodities are managed by the MOH through the National Drug Service Organization (NDSO) and commodities are frequently out of stock, leading to service interruptions, which may hinder integration. Additionally, human resource issues were discussed including staff shortages and the high turnover rate as a potential barrier to integration, with one respondent noting:

“*We need consistent training of service providers because we encounter high turnover of health providers*.”

-Implementing Partner

Key informants provided some suggestions to improve service readiness, such as the use of mobile clinics, which are able to reach rural and underserved populations, as an existing service delivery platform which would be ideal for providing integrated services. Key informants also suggested implementing community education and demand creation programs. One respondent summarized the need for demand creation well, stating:

“*Demand creation is very critical […] We need our clients to come already knowing so they are able to ask for services*.”

-Implementing Partner

Providers reported some demand creation activities at their sites in the form of client education sessions in the waiting areas and the provision of educational materials for clients on FP methods and/or HIV prevention. At one community site, educational materials were available and educational sessions for clients were conducted by peer educators. A different community site also reported having some educational materials available. At the facility level, two FP providers said that they have FP posters in the waiting area and educational sessions are conducted. The remaining provider reported that while there are no materials for clients to read, all staff at their facility are responsible for conducting educational sessions. However, five FP providers (three at the community level and two at the facility level) reported that they do not have such sessions or materials.

Additional areas for improved service readiness mentioned by key informants were developing Monitoring and Evaluation (M&E) tools that accommodate PrEP/FP integrated services, implementing flexible working hours to accommodate clients with non-traditional schedules, such as factory workers and AGYW, and having guidelines and job aids to support provision of both FP and PrEP. Finally, the general need for coordination among implementing partners in order to avoid duplication of efforts and effectively monitor programs was identified as an essential need.

#### FP service provision

We explored current availability of FP methods as well as client preferences/method utilization in order to consider how/if site visits for PrEP and FP methods can be synchronized. Family planning providers reported that contraceptive method mix at the community level sites is limited to short-acting methods, including OCPs, progestin-only injectables, and condoms. Clients who desire a method that is not available on site, such as the intrauterine device (IUD) or contraceptive implant, are referred to the nearest facility with the capacity to provide this method (either a LPPA facility or a government hospital); however, community-level providers noted that they are not able to track FP referrals to ensure completion. At the facility level sites, in addition to the same selection of short-acting methods, providers offered contraceptive implants, and in some, but not all facilities, IUDs. FP services at both the community- and facility-level are provided free of charge with the exception of pregnancy tests, if conducted to rule out pregnancy prior to initiating contraceptive methods.

With regards to client preferences, all community-level providers said that AGYW clients prefer OCPs, while two providers mentioned that some young clients choose injectables due to convenience and concerns about being able to adhere to daily OCPs use. Facility-level providers said that many of their clients prefer injectables due to the less frequent need for clinic visits, the ability to use this method without their partner's knowledge, and the efficacy, stating:

“*Injectables will never disappoint them and [they will not] fall pregnant unexpected*.”

-Facility-level provider

Facility-level providers stated that implants are also popular with FP clients, though due to frequent stock-outs, clients often select injectables instead. Condoms were often chosen for their ability to protect from sexually transmitted infections (STIs) in addition to pregnancy. Among young clients, condoms were often chosen because they can be used without their parents' knowledge. However, providers noted that condom use remained limited by stigma and misconceptions, including the perception that married couples have no need for condoms, and concerns about negotiating condom use, particularly among AGYW.

All 10 providers noted that stock-outs of contraceptive methods, particularly OCPs, injectables, and implants, were common and lasted a short period of time approximately every 3 months. A provider stated:

“*We face constant stock-out of the oral contraceptive pills because people prefer them more than the others and [the] 2-month injectable because of its demand*.”

-Community-level provider

One reason given for stock-outs were challenges with keeping up with increasing demand, as orders for resupply are calculated based on the usage data form the previous month.

Three providers experienced staff shortages at their facilities, with some being able to offer FP only on one particular day of the week, and one of the facilities having only one provider able to provide IUDs, who was not always available. One provider summarized the staffing shortage as follows:

“*We do not have adequate staff because we serve many people but there are only two of us in here; when one is absent, the one on duty works very hard*.”

-Facility-level provider

Most community-level providers noted that they receive regular supportive supervision. This was not the case with facility-based providers, with four out of five reporting limited supervisory support with providers stating that “*supportive supervision is not adequate*” and “*there is no supervision*.”

Outreach services for underserved populations was done by community-level providers only, while facility-level providers felt that due to a sufficient number of facilities in the district, they are easily reachable by clients and no outreach was needed.

#### HIV service provision

At the facility level, three out of five FP providers were not involved in offering HIV services directly but did so through referral to an HIV provider within their facility. One other facility-based FP provider offered ART, post-exposure prophylaxis (PEP), and PrEP (even though this provider reported receiving no formal training on PrEP), while another provided just ART in addition to FP. While HIV services were often provided in a different location within the facility and not necessarily by the FP provider, facility-level providers considered their services integrated and described it as a “supermarket approach” where all necessary services are available at the facility on the same day though frequently in different departments. Community-level providers all offered HIV testing and/or assistance with self-testing, pre-and post-test counseling, and referrals for other services, such as ART, PEP, and PrEP, depending on clients' needs and preferences. Risk assessment for STIs and HIV was performed by all 10 providers; however, facility-based providers noted that they lack clear guidance on how to conduct the assessment and would benefit from having risk assessment tools and/or job aids. Condoms were universally recommended by all providers as part of dual protection from both pregnancy and STIs, including HIV.

At the facility level, all providers reported that PrEP services were available multiple days a week, but usually from a different location within the facility, most commonly from the ART corner. While PrEP referrals within the facility are not formally tracked, three FP providers said that they often take their clients directly to a PrEP provider to avoid having them wait in another queue. At the community level, PrEP was available only through referrals to a facility, which is commonly located in the building next to the tent providing community-level services. A provider discussed the process of facilitating PrEP services for clients:

“*Clients needing both these services get services at the same day. When she finishes in here, I ensure that she arrives at PrEP corner by accompanying her, and to make sure that she does not queue since she got here before those on the line. She stands behind the 1st person on the queue*.”

-Facility-level provider

In addition to referrals, all community-based providers said that they educate their clients about PrEP availability. Only one community-level provider said that they have a system in place to track PrEP referrals.

### Service utilization and willingness to use PrEP

#### FP clients who would consider using PrEP

Six FP clients in the sample used injectable contraception, split evenly between NET-EN and depot-medroxyprogesterone acetate (DMPA), and one client used OCPs. Clients stated that they were able to obtain their desired method of contraception in some cases, but in others, they reported stock-outs impeding method choice. Some clients discussed being denied their method of choice due to body weight, menstruation requirements, or pregnancy test requirements, all of which are medical eligibility barriers. All clients said that the facilities they used were accessible as approximate mean travel time was 20 mins by vehicle for some and walking for others.

Satisfaction with services from FP providers was universal among the clients interviewed. They felt that providers protected confidentiality, allowed for an open dialogue to ask questions and discuss concerns, and generally were well liked. A client described her experience with her FP provider as follows:

”*I liked their approach; it was very friendly and made it easy for me to communicate with them and ask questions where needed.“*

-FP client at community level, 21-years-old, single with no children

Clients also expressed satisfaction with the way FP services are structured. Most clients we interviewed stated that they receive FP services and commodities free of charge, though costs were associated with pregnancy tests which some providers required before providing a refill or reinjection if a visit was missed. FP clients also discussed that the queue and wait time for services was shorter than they experienced with other services. A client explained:

“*And the waiting time is bearable. There are no long queues.”*

*-* FP client at community level, 21-year-old, single with no children

Stigma and disclosure of FP use to partners was explored and most clients had disclosed to their partners that they used FP and the partners supported their use. One client described her partner's support:

“*I don't need to keep my FP use private. My partner doesn't have any problem. He supports me [and he] reminds me of my check-up date.”*

-FP client at facility level, 28-year old, married with 1 child

Two clients had not disclosed FP use to their partners with one client explaining that she had not disclosed because the use of FP was solely her decision.

The level of knowledge of PrEP among the FP clients in the assessment was explored and responses varied among clients interviewed. All of them were aware that they could obtain PrEP from a facility and had a general awareness that PrEP is taken by uninfected people to prevent HIV infection, as is illustrated in this quote:

“*PrEP is used for HIV-negative people who engage in sexual activities with people they do not know their HIV status or someone with a positive HIV status.”*

-FP client at facility level, 26-years-old, married with 1 child

Two FP clients reported a comprehensive understanding of PrEP, including when and how to take the medication. The other FP client in the sample demonstrated less of an understanding about PrEP as they were unclear how often to take PrEP, the length of time the medication needs to be taken for, and when the medication may be discontinued. Additionally, two FP clients mistook PEP for PrEP.

In terms of current/past PrEP use and willingness to use it, one FP client is currently taking PrEP and would like to continue taking it indefinitely, and the remaining six clients have never used the medication. When clients were asked for their opinions on whether they believed women in the community would consider using PrEP, they thought that women would likely use PrEP if given the opportunity, as noted in the below quote.

”*I think [women would use it] because people encounter many challenges in life, and they might use it to avoid being infected.“*

-FP client at facility level, 28-years-old married with 1 child

Multiple FP clients questioned partner faithfulness and felt that PrEP would be taken up by women as a way to protect themselves against acquiring HIV. Clients believed that the largest barrier to PrEP uptake in the community is the lack of knowledge about PrEP (including misconceptions) and noted that community education was needed. Clients were asked if they felt that stigma or intimate partner violence would be a concern if they decided to use PrEP. The majority stated that they felt their partner and the community would support their PrEP use. A community-based client described her perspective on disclosure in general:

“*There would be no need for me to keep my PrEP use a secret; I would be proud just like my friends who use it because I will be protecting myself.”*

-FP client at community level, 21-years-old single with no children

The same client, who is in a relationship, explained her thoughts on disclosing use to her partner.

”*I think my partner will not have a problem with me using PrEP on top of family planning because he will know it is not because I do not trust him, but it is because I take care of myself and look after my health because we all know that HIV can be transmitted in many different ways.“*

-FP client at community level, 21-years-old single with no children

#### PrEP clients who are using FP or would consider future FP use

Seven out of the eight PrEP clients were also using FP and all were able to receive their preferred method during their initial visit—three were using injectables (either NET-EN or DMPA), three were using OCPs, and one was using the two-rod levonorgestrel implant (Jadelle)—which they access from various locations based on where they are when they need their refill or reinjection. Four clients stated that they last received FP services from the local community center and the remainder went to the university clinic, facility, or the pharmacy. One client preferred seeking FP services at the pharmacy, despite the cost, in order to save time and not have to wait in the long queue at the facility. Two other clients discussed COVID-19 lockdown disruptions to their FP services, stating that they were unable to receive their usual free services from the community center and had to instead buy their FP from the pharmacy. One facility-level client mentioned experiences with provider misconceptions around menses requirements for refills as a barrier to care though she was able to switch to community-level services and receive desired care. Two respondents also mentioned experiencing method stock-outs, which necessitated method switching.

The PrEP clients interviewed were generally well informed about PrEP and how it works, with all respondents understanding that PrEP prevents HIV infection. One respondent described PrEP as follows:

“*PrEP is a pill that a person can use when they feel like they are at risk of being infected with HIV and it is taken by everyone as long as they are HIV-negative, but you can stop when you feel like you are no longer at risk.”*

-PrEP client at community level, 21-years-old, single with no children

The majority of PrEP clients mentioned that PrEP reduces the chance of HIV infection by 90% and it can be stopped whenever one feels they are no longer at risk of acquiring HIV. One client mentioned certain steps to follow to stop taking PrEP:

”*When you feel like you are no longer at risk, you can stop taking it. You don't just stop using it; you take some time after having unprotected sex, not sure how many days. Twenty-one days.“*

-PrEP client at community level, 22-years-old, single with no children

However, another client from the facility level was not counseled about when PrEP can be stopped and assumed it should be taken indefinitely.

Two of the clients did mention characteristics of PrEP that are misconceptions. One of them said that PrEP may prevent HIV infection if taken close to the time of sexual intercourse, which is currently recommend by the WHO only for men who have sex with men. Another client stated:

”*Because I was pregnant, I was told that PrEP will help strengthen my placenta.”*

-PrEP client at facility level, 39-years-old, married with 4 children

All clients indicated that for each refill, they access PrEP at the same site. All respondents that indicated their date of PrEP initiation had started within the past 5 years. When asked why they started PrEP, five clients said they are in serodiscordant relationships, and three were taking PrEP because they did not trust their sexual partners. All clients reported being able to access the facility or community site in less than an hour by foot or transport (20–45 minutes travel).

All clients expressed desire for multi-month dispensing of PrEP to decrease the amount of health care visits required and reduce transportation cost, though only one client noted that she was able to get a 3-month supply at her last refill. A few clients discussed that they are able to get a 3-month supply sometimes, though it depends on the availability of PrEP stock at the time. One respondent explained that when PrEP stocks were running low at the facility, the provider will give her a few days' supply of PrEP pills to last until the full refill can be issued, which then creates the appearance of non-adherence:

“*You will find that they give pills for seven days, and then when I come back, I get my usual three-month supply, but they do not take out the seven pills I have already taken, so we have issues of adherence because next time they think that I haven't taken all the pills*.”

-PrEP client at facility level, 30-years-old, married, 1 child

Clients indicated that the time needed to receive PrEP services at facilities or PrEP sites differs depending on the type of visit. For instance, they highlighted that PrEP initiation visits took anywhere from 15 to 40 minutes and included counseling, while refill visits were very quick. After arriving at the facility, waiting times varied as queues are generally short early in the day.

Satisfaction with PrEP use was high among clients reporting feeling reassured that they are protected against HIV and with none reporting any side effects. One client stated:

“*What I like about taking PrEP is that I am no longer stressed because of not trusting my partner. I have a peace of mind.”*

- PrEP client at community level, 21-years-old, single with no children

Six of the eight PrEP clients interviewed expressed the desire to continue using PrEP either indefinitely or until they felt they no longer needed HIV protection, with two clients further explaining:

“*It makes me comfortable in my marriage because when I got married, I did not know that my husband was HIV-positive, so as a result was scared of what was going to become of me, and now, I am happy I will be able to live with someone I love.”*

- PrEP client at facility level, 39-years-old, married with 4 children

“*I like taking PrEP because I am safe from getting infected with HIV, and that I am going to stop taking it once I feel I am no longer at risk. It's not a lifetime pill. It stops the spread of HIV.”*

- PrEP client at community level, 22-years-old, single with no children

All eight PrEP clients noted that they were highly satisfied with the manner in which PrEP services are provided. They touched on the personality and attitude of PrEP providers, describing providers as open, welcoming, and friendly. Clients were happy with the free PrEP services and the majority felt that the quality of service provided was high with privacy and confidentiality being maintained. Privacy concerns were expressed by one respondent, as they felt that the provision of PrEP services within the designated ART corner may lead others to assume that the client is living with HIV. Seven clients indicated that the services are offered at convenient times; however, one client, who is a factory worker, said that PrEP services are not convenient because they are only offered during working hours on weekdays, so she has to take off work to access them.

All married clients reported disclosing their PrEP use to their partners, and that their partners were supportive of them. However, the two unmarried clients stated they had not disclosed their PrEP use to their partners, but felt that they would have their partner's support if they were to disclose. Family disclosure and support was also high, with five clients describing strong parental and mother-in-law support of PrEP use. Some clients decided not to disclose to their family and keep their PrEP pills hidden, as one client stated:

“*In the presence of family members, we keep them secretly in a kitchen unit where they won't see them.”*

- PrEP client at facility level, 22-years-old, married with 1 child

## Discussion

Evidence on integrating PrEP into FP services is limited, as PrEP is a fairly new HIV prevention approach and not yet widely available in Sub-Saharan Africa through service delivery platforms other than HIV treatment and care services (8–12). This assessment was designed to add to the evidence base through a qualitative exploration of the feasibility and acceptability of integrating PrEP into FP services. Four key themes were identified: (1) the enabling environment, which highlighted policy and programmatic facilitators and barriers, (2) perceptions of acceptability of integrated services which, showed both preference for and concerns about integrated services, (3) service readiness which highlighted current service delivery strengths and areas for improvement, and (4) service utilization and willingness to use PrEP, which showed current use of PrEP and FP services as well as challenges to be addressed. Across these four themes, key barriers to and facilitators of integrated services were identified by all categories of respondents (Table 2).

**Table 2:** Barriers and facilitators to integrating PrEP into FP services by theme.

**Theme**	**Integration facilitators**	**Integration barriers**
Enabling environment	•Political commitment and increase in funds allocation toward commodities procurement (KI)	•Lack of policies guiding integration of PrEP into FP services (KI)
	•National pool of trainers is available and can offer in-service training on PrEP (KI)	•Siloed funding of FP and HIV services (KI)
		•Lack of training on PrEP among FP providers (KI, FBP)
		•Lack of job aids and guidelines (FBP, CBP)
		•Commodity stock-outs (KI, FBP, CBP, FBC, CBC)
		•Separate registers and reporting system for FP and PrEP (FBP, CBP)
		•Structural separation of PrEP and FP within Ministry of Health departments and lack of technical working group that shares information on PrEP and FP (KI)
		•Insufficient infrastructure and commodity stock-outs (KI, FBP, CBP)
Perception of acceptability	•Perception that PrEP integration will be well received by those in need of FP services (KI, FBP, CBP)	•Concern about increase in waiting time (KI, CBP)
	•Perception that integration will improve continuation and adherence, and reduce incomplete referrals (FBP, CBP)	•Concern about information overload (CBP, KBP)
	•Preference for getting both services—PrEP and FP—in a single visit from a single provider, even if it means longer wait time (FBC, CBC)	•Misconceptions about PrEP within communities, including confusing PrEP with regular ART (FBC) and associating PrEP use with promiscuity (KI)
Service readiness	•Some familiarity with integrating other components of HIV prevention, such as HIV counseling and testing, STI/HIV risk assessment, and dual protection counseling (CBP, FBP)	•Staff shortages, high staff turnover, heavy workloads (KI, FBP, CBP)
	•Plans to develop guidelines and job aids in support of FP/PrEP integration (KI)	•Burden of multiple reporting forms (KI)
	•Implementing flexible work hours (KI)	•Limited access to FP services in some areas (KI)
	•Some demand creation and education materials and activities currently available at sites (CBP, FBP)	•Limited supportive supervision (FBP)
		•Concern about effects of the increased workload on service quality (CBP, FBP)
		•Stockouts of FP and PrEP (CBP, FBP)
Service utilization and willingness to use PrEP	•General awareness that PrEP is available and can be used to prevent HIV infection (CBC, FBC)	•Limited multi-months dispensing of PrEP (CBC, FBC)
	•Partner support for PrEP use (CBC, FBC)	•Concern about not being able to align visits for PrEP refills with FP method refill/re-injection (CBC, FBC)
	•Reduced number of visits, savings in terms of time and transportation costs (CBC, FBC, CBP, FBP)	•Lack of knowledge about PrEP within communities; need for outreach and education (CBC, FBC)
	•Satisfaction with providers (CBC, FBC)	

In parenthesis, type of the respondent who mentioned a particular barrier or facilitator is identified as follows: KI, key informants (including implementing partners, policy makers, and funders); FBP, facility-based providers; CBP, community-based providers; FBC, facility-based clients; CBC, community-based clients.

It is clear that there is government support for PrEP and FP services with a substantial amount of domestic funding being allocated to both ARVs/PrEP and FP commodities. This makes Lesotho uniquely positioned to provide sustainable integrated services as its reliance on donor funding for both FP commodities and ARVs (including PrEP) is significantly lower than in most low- and middle-income countries (19, 20). The availability of a pool of national trainers who can offer in-service training on PrEP to FP providers is another key facilitator within the enabling environment. Despite these facilitators, we found multiple high-level barriers to integration including the lack of explicit policies and guidelines supporting PrEP integration into FP services, siloed funding mechanisms, lack of coordination between HIV and FP MOH departments, limited MOH ownership of HIV interventions that are highly dependent on implementing partners, and perception that services are already being integrated through the “supermarket approach.” Staffing challenges including the availability and heavy workload of trained staff also was discussed by providers and key informants who expressed concerns that it may be hard to take on any additional tasks for providers who are already working at understaffed facilities. Moreover, providing integrated services requires both FP commodities and PrEP being readily available; however, this assessment found that, in spite of government commitment, stock-outs of both PrEP and FP methods, such as OCPs, injectables and implants are common, which will not only hamper integrated service provision but also prevent multi-month dispensing, despite supportive policy around this practice.

Findings from this assessment show that support for integrating PrEP into ongoing FP programming is generally high, especially among clients who have a strong preference for receiving comprehensive services from one provider at a single visit. Client support for integrated services is mainly driven by the desire to save money and time by reducing the number of health care visits—a finding that is supported by the literature (4, 21). Providers also considered time and money benefits to clients, and also discussed the potential for integrated services to improve FP continuation and PrEP adherence. Though there is not yet enough data on these potential outcomes, prior studies and programs in Kenya and South Africa that have looked at integrating services have found some success in PrEP uptake and continuation achieved with well-trained providers, program-dedicated staff, and strong government support (8–10). Though acceptability of integrated services was high in our assessment, research has shown that PrEP-related stigma can be a driver of lower uptake (22). Our respondents also highlighted the need for educating communities about PrEP, promoting greater understanding of the benefits of PrEP, and addressing stigma and misconceptions surrounding its use, such as associating PrEP use with promiscuity and confusing PrEP with regular ART.

In terms of service readiness, provider training on PrEP was highlighted as a key component to integrated service provision. It is encouraging that many of the providers interviewed were either already providing HIV services other than PrEP or had some familiarity with components of HIV prevention, which may serve as foundation for PrEP training. Some sites reported already having demand creation activities in place such as group health talks in waiting areas and educational materials such as posters and brochures. Expanding on ongoing activities to include information on both PrEP and FP may potentially serve to increase awareness and thus demand for integrated services at sites. However, staff shortages, high staff turnover, and heavy existing workload was discussed as a key factor hindering readiness to provide integrated services. Remedying these challenges will require resources and government commitment to hire, train, and retain trained staff. Additionally, the need for supportive supervision was discussed, which is currently limited, but will be particularly important in order for integrated services to succeed.

The final major theme identified, service utilization and willingness to use PrEP, was explored by clients. Among clients using PrEP, partner support was high, and most clients who were not using PrEP felt that their partners also will be supportive of their decision. This finding is encouraging as partner support has been shown to be important in both PrEP initiation and continuation (23–25). Both current PrEP clients and FP clients displayed general awareness of PrEP as a tool for HIV prevention; however, both types of clients expressed concern that the community at large did not have basic knowledge and understanding about PrEP and needed outreach and education in order to encourage PrEP use.

We explored FP method preferences and utilization in order to understand opportunities for synchronizing refills. Our findings indicate that while OCPs and injectable contraceptives, which are the methods that are preferred by a majority of FP clients, allow for aligning PrEP refills with OCP refills or DMPA reinjection, some barriers still exist. One barrier mentioned was the lack of alignment between refill schedules for PrEP and some other methods, such as the popular injectable, NET-EN, which is given every 2 months while PrEP is usually dispensed at a 3-months intervals (although it heavily depends on its availability). The lack of alignment of refill schedules can also occur inadvertently due to stockouts of PrEP and/or FP. This issue can be addressed through adjusting resupply schedules and maintaining reliable commodity stocks; however, both solutions require addressing larger systemic issues such as the supply chain and clinical guideline revisions to allow for greater flexibility.

The findings of this assessment highlight multiple barriers and we consider the following as the key ones that need to be addressed in order to effectively implement integration of PrEP into FP services. First, policies and guidelines, especially national FP guidelines, need to explicitly address PrEP/FP integration, so providers have the guidance and tools to implement integrated service delivery. Next, a successful integration of PrEP needs input from both HIV and FP partners (particularly both departments within the MOH) in the form of a joint HIV/FP Technical Working Group to facilitate planning and coordination. Third, the donor community should consider structuring funding in a way that avoids vertical silos and allows for integrated programming. To successfully deliver integrated services at the facility level, FP providers need training and tools, including in-service training on PrEP, and job aids supporting PrEP counseling, eligibility screening and provision. In addition, providers need structural and policy support including coordinated supply chains, alignment (as much as possible) of FP return visits with the PrEP re-supply schedule, and integration of the reporting registers so they include information on both FP and PrEP. And finally, community outreach and sensitization/education about PrEP and its availability within FP services should be a vital part of any integration efforts to ensure that clients who may be at risk of both pregnancy and HIV are aware of and can fully benefit from dual prevention opportunities

## Limitations

The purpose of this assessment was to generate information that could help shape future research, inform policy decision making, and/or improve program implementation. While the results are not generalizable, the assessment reflects the perceptions of a range of stakeholder groups and provides valuable, actionable insights on the feasibility and acceptability of integrating PrEP into FP services in Lesotho. The assessment may also help to formulate additional implementation research questions to inform integrated programs. Key limitations of this assessment were the geographic restriction to Maseru District and use of convenience sampling, which may have introduced selection biases. Additionally, IDI sample sizes were not large enough to reach 80% saturation, as recommended in order to identify the most important themes of a study (17, 18). Lastly, the assessment was limited by the inability to conduct all interviews in person as initially planned due to COVID-19 related restrictions. However, the assessment team does not feel that data collection done on the phone impacted the quality of data obtained.

## Conclusion

As PrEP becomes more widely available in Sub-Saharan Africa, making this effective, user-controlled HIV prevention approach available from health providers outside of the HIV sector, such as FP providers, may lead to more equitable access to and use of services and help populations that may be at risk of acquiring HIV make informed choices about both their HIV and pregnancy prevention options. This assessment provides valuable information for the implementation of PrEP integration into FP services that can help guide future policies and programming, and inform additional research within and outside of Lesotho.

## Data availability statement

The qualitative data generated and analyzed during the current study are not publicly available for ethical reasons because even after removing directly identifiable information such as names, participant identities may be difficult to fully conceal and research locations may remain potentially identifiable, presenting a risk of deductive disclosure. The data collection instruments, informed consent forms, and recruitment script are available here https://doi.org/10.7910/DVN/UIXLKT.

## Ethics statement

The studies involving human participants were reviewed and approved by FHI 360's Protection of Human Subjects Committee, Johns Hopkins School of Public Health Institutional Review Board, Lesotho Ministry of Health Research and Ethics Committee. Written informed consent for participation was not required for this study in accordance with the national legislation and the institutional requirements.

## Author contributions

EL, N-MN, MMat, IY, MS, MR, MMal, and TC contributed to the conception and design of the assessment and interpretation of study results. EL, N-MN, MMat, IY, and TC performed the analysis. EL, N-MN, MMat, and IY wrote the first draft of the manuscript. All authors contributed to the manuscript revisions, read, and approved the submitted version.

## Funding

This work is made possible by the generous support of the American people and the U.S. President's Emergency Plan for AIDS Relief (PEPFAR), through the U.S. Agency for International Development (USAID), provided to FHI 360 through Cooperative Agreement AID-OAA-A-15-00045.

## Conflict of interest

The authors declare that the research was conducted in the absence of any commercial or financial relationships that could be construed as a potential conflict of interest.

## Publisher's note

All claims expressed in this article are solely those of the authors and do not necessarily represent those of their affiliated organizations, or those of the publisher, the editors and the reviewers. Any product that may be evaluated in this article, or claim that may be made by its manufacturer, is not guaranteed or endorsed by the publisher.

## Author disclaimer

The contents are the responsibility of FHI 360 and do not necessarily reflect the views of USAID or the United States Government.

## References

[1] Leostho Population-Based HIV Impact Assessment. LePHIA 2020: Summary Sheet. (2020). Available online at: https://phia.icap.columbia.edu/lesotho-summary-sheet-2/ (accessed July 2021).

[2] UNAIDS. AIDSInfo. (2020). Available online at: http://aidsinfo.unaids.org/ (accessed March 15, 2022)

[3] Fonner VA Dalglish SL Kennedy CE Baggaley R O'Reilly KR Koechlin FM. Effectiveness and safety of oral HIV preexposure prophylaxis for all populations. AIDS. (2016) 30:1973–83. 10.1097/QAD.0000000000001145 27149090 PMC4949005

[4] Church K Mayhew SH. Integration of STI and HIV prevention, care, and treatment into family planning services: a review of the literature. Stud Fam Plann. (2009) 40:171–86. 10.1111/j.1728-4465.2009.00201.x 19852408

[5] Narasimhan M Yeh PT Haberlen S Warren CE Kennedy CE. Integration of HIV testing services into family planning services: a systematic review. Reprod Health. (2019) 16:61. 10.1186/s12978-019-0714-9 31138307 PMC6538541

[6] Warren CE Mayhew SH Hopkins J. The current status of research on the integration of sexual and reproductive health and HIV services. Stud Fam Plann. (2017) 48:91–105. 10.1111/sifp.12024 28493283 PMC5518217

[7] Drake AL Quinn C Kidula N Sibanda E Steyn P Barr-DiChiara M. A landscape analysis of offering HIV testing services within family planning service delivery. Front Reprod Health. (2021) 3:657728. 10.3389/frph.2021.657728 PMC9580748 36304029

[8] Kasaro MP Sindano N Chinyama M Mudenda M Chilaisha F Price JT. Integration of HIV prevention with sexual and reproductive health services: evidence for contraceptive options and HIV outcomes study experience of integrating oral pre-exposure HIV prophylaxis in family planning services in Lusaka, Zambia. Front. Reprod. Health. (2021) 3:684717. 10.3389/frph.2021.684717 PMC9580744 36304051

[9] Mansoor LE Yende-Zuma N Baxter C Mngadi KT Dawood H Gengiah TN. Integrated provision of topical pre-exposure prophylaxis in routine family planning services in South Africa: a non-inferiority randomized controlled trial. J Int AIDS Soc. (2019) 22:e25381. 10.1002/jia2.25381 31507088 PMC6737288

[10] Mugwanya KK Kinuthia J. Integrating pre-exposure prophylaxis delivery in public health family planning clinics: lessons learned from a programmatic implementation project in Kenya. Front. Reprod. Health. (2021) 3:683415. 10.3389/frph.2021.683415 PMC9580668 36304007

[11] Mugwanya KK Pintye J Kinuthia J Abuna F Lagat H Begnel ER. Integrating preexposure prophylaxis delivery in routine family planning clinics: a feasibility programmatic evaluation in Kenya. PLoS Med. (2019) 16:e1002885. 10.1371/journal.pmed.1002885 31479452 PMC6719826

[12] High Awareness Y. of Pre-exposure prophylaxis among adolescent girls and young women within family planning clinics in Kenya. AIDS Patient Care STDS. (2020) 34:336–43. 10.1089/apc.2020.0037 32757980 PMC7415219

[13] Ministry Ministry of Health [Lesotho] ICF International. Lesotho Demographic and Health Survey 2014. (2016). Maseru, Lesotho: Ministry of Health and ICF International.

[14] Lesotho Lesotho Ministry of Health Government Government of Lesotho. Addendum to the National Guidelines on the Use of Antiretroviral Therapy for HIV Prevention and Treatment. (2019). Lesotho: Lesotho Ministry of Health, Government of Lesotho.

[15] Lesotho Ministry of Health. Lesotho National Family Planning Guidelines for Health Service Providers. (2017). Lesotho: Lesotho Ministry of Health.

[16] Bhavaraju N Wilcher R Regeru RN Mullick S Mahaka I Rodrigues J. Integrating oral PrEP into family planning services for women in Sub-saharan Africa: findings from a multi-country landscape analysis. Front. Reprod. Health. (2021) 3:667823. 10.3389/frph.2021.667823 PMC9580806 36303993

[17] Guest G Bunce A Johnson L. How many interviews are enough? An experiment with data saturation and variability. Field Methods. (2006) 18:59–82. 10.1177/1525822X05279903

[18] Namey E Guest G McKenna K. Evaluating bang for the buck: a cost-effectiveness comparison between individual interviews and focus groups based on thematic saturation levels American journal of evaluation. 37:425–40. 10.1177/1098214016630406

[19] Center for Global Development Kaiser Family Foundation. The UST International Family Planning Landscape: Defining Approaches to Address Uncertainties in Funding and Programming. (2018). Available online at: https://files.kff.org/attachment/Issue-Brief-The-USG-International-Family-Planning-Landscape-Defining-Approaches-to-Address-Uncertainties-in-Funding-and-Programming (accessed August 5, 2022).

[20] Kaiser Family Foundation. Funding for Key HIV Commodities in PEPFAR Countries. (2021). Available online at: https://www.kff.org/report-section/funding-for-key-hiv-commodities-in-pepfar-countries-issue-brief/ (accessed August 5, 2022).

[21] Colombini M Mayhew SH Mutemwa R Kivunaga J Ndwiga C. Perceptions and experiences of integrated service delivery among women living with HIV attending reproductive health services in Kenya: a mixed methods study. AIDS Behav. (2016) 20:2130–40. 10.1007/s10461-016-1373-2 27071390 PMC4995223

[22] Golub SA. PrEP stigma: implicit and explicit drivers of disparity. Curr HIV/AIDS Rep. (2018) 15:190–7. 10.1007/s11904-018-0385-0 29460223 PMC5884731

[23] Gombe MM Cakouros BE Ncube G Zwangobani N Mareke P Mkwamba A. Key barriers and enablers associated with uptake and continuation of oral pre-exposure prophylaxis (PrEP) in the public sector in Zimbabwe: qualitative perspectives of general population clients at high risk for HIV. PLoS ONE. (2020) 15:e0227632. 10.1371/journal.pone.0227632 31931514 PMC6957335

[24] Bärnighausen K Geldsetzer P Matse S Hettema A Hughey AB Dlamini P. Qualitative accounts of PrEP discontinuation from the general population in Eswatini. Cult Health Sex. (2021) 23:1198–214. 10.1080/13691058.2020.1770333 32633617

[25] Bjertrup PJ Mmema N Dlamini V Ciglenecki I Mpala Q Matse S. PrEP reminds me that I am the one to take responsibility of my life: a qualitative study exploring experiences of and attitudes towards pre-exposure prophylaxis use by women in Eswatini. BMC Public Health. (2021) 21:727. 10.1186/s12889-021-10766-0 33853575 PMC8048211

